# Benzodiazepine use, quality of life and psychiatric symptom burden in oral and injectable opioid agonist treatment: a cross-sectional study

**DOI:** 10.1186/s13722-023-00397-8

**Published:** 2023-07-18

**Authors:** Maximilian Meyer, Ferdinand Gygli, Jean N. Westenberg, Otto Schmid, Johannes Strasser, Undine E. Lang, Kenneth M. Dürsteler, Marc Vogel

**Affiliations:** 1 University of Basel Psychiatric Clinics, University of Basel, Wilhelm Klein-Strasse 27, 4002 Basel, Switzerland; 2 Cantonal Hospital Baden, Baden, Switzerland; 3 Department for Psychiatry, Psychotherapy and Psychosomatic, Psychiatric Hospital, University of Zurich, Zurich, Switzerland

**Keywords:** Opioid use disorder, Quality of life, Methadone maintenance treatment, Heroin-assisted treatment, Substitution treatment, Sedative

## Abstract

**Background:**

Use of benzodiazepines (BZD) in patients receiving opioid agonist treatment (OAT) is common and associated with a variety of negative health and social outcomes. This cross-sectional study investigates the impact of BZD use in OAT patients on their quality of life (QoL).

**Methods:**

A convenience sample of patients receiving oral OAT or heroin-assisted treatment in two outpatient centres in Basel, Switzerland was investigated. Participants (n = 141) completed self-report questionnaires on psychiatric symptoms and psychological distress (The Symptom Checklist 27, SCL-27), depressive state (German version of the Center for Epidemiological Studies Depression Scale), quality of life (Lancashire Quality of Life Profile, LQOLP) and use of BZD and other drugs (self-report questionnaire). Substance use was assessed by urine toxicology testing.

**Results:**

In bivariate analysis, total QoL scores were significantly lower for lifetime, current, and prolonged BZD users compared to participants without the respective use patterns. There was no significant relationship between BZD dose and QoL. In multivariable linear regression models controlling for psychiatric symptom load and depressive state, only lifetime use predicted lower QoL, whereas other BZD use patterns were not significantly associated.

**Conclusions:**

The association of lower QoL and BZD use in OAT patients is strongly confounded by co-occurring depressive state and psychiatric symptoms. Careful diagnosis and treatment of co-occurring mental disorders in OAT is paramount to improve QoL in this patient population and may also help reduce BZD use.

## Background

Benzodiazepines (BZD) are widely used psychoactive drugs, mostly prescribed to manage anxiety and affective disorders, insomnia, alcohol withdrawal, delirium as well as agitation, aggression, and violent behaviour in psychosis [1]. Concurrent prescription and non-prescription use of BZD is common among patients with opioid use disorder (OUD) receiving opioid agonist treatment (OAT). The point prevalence in the literature ranges from 15 to 50.8% [2–5], whereas lifetime prevalence is estimated between 47 and 85% [2, 6, 7]. However, use of BZD in OAT is associated with a variety of negative health and social outcomes. These include a higher risk of unemployment, imprisonment, loss of libido, continued use of illicit substances, overdose-related death, and higher psychopathological and emotional distress [4, 8–11]. Furthermore, concurrent BZD use in OAT patients is associated with a reduction in quality-of-life (QoL) [12, 13]. QoL is an important patient-related outcome, as individuals with OUD generally report lower average QoL compared to the general population. Participation in OAT among patients with OUD generally improves self-perceived health and QoL, indicative of improved overall well-being [14].

Motives for BZD use in OAT can be categorised in negative affect regulation (e.g., to manage anxiety), positive affect regulation (e.g., to get or enhance a high), and somato-medical motives (e.g., to regulate sleep or manage withdrawal symptoms) [6, 15–17]. BZD use for negative affect regulation as well as somato-medical motives likely resembles a maladaptive coping strategy, in line with the self-medication theory [18]. Along with the finding that the onset of OUD is often preceded by anxiety disorders, this possibly explains the high BZD use prevalence observed in this population [19]. Furthermore, BZD may be prescribed as an off-label maintenance approach in patients with comorbid BZD dependence [20].

Evidence on the relationship between OAT retention and BZD use is inconclusive. Franklyn et al. (2017) found that OAT patients with concurrent BZD use are 15% more likely to terminate treatment prematurely, whereas other scholars found concurrent BZD prescriptions to be associated with increased duration of OAT [21, 22]. These contradictory findings might be explained by the observation that patients sometimes learn how to use BZD as prescribed over the course of treatment. Whereas BZD use is more likely to start as BZD misuse in the initial OAT phase (e.g., to get a high), they are often used as intended in later treatment stages (e.g., to treat anxiety) leading to patients experiencing positive outcomes [23].

The association of BZD use with QoL, co-occurring mental disorders, psychiatric symptom load, and psychological distress has not yet been thoroughly investigated in OAT patients. Moreover, there are no studies on patients in injectable OAT, who may be at a higher risk for BZD-related adverse effects such as overdose. In non-opioid-dependent individuals, significant BZD dose decreases are associated with improvements in self-rated QoL [24]. However, following the implications of the self-medication theory, lower BZD use might also be associated with higher psychiatric symptom load, therefore impairing QoL. Investigating this issue may have clinical implications for the use of prescription BZD in OAT patients. As well, it contributes to a better understanding of the complex relationship between BZD use, QoL and psychiatric symptoms among patients receiving OAT.

## Methods

### Study aim, design and setting

This study aimed to answer the following two research questions: (1) Are there observable differences between BZD use patterns and QoL among OAT patients and (2) Which treatment and non-treatment-related factors influence the association between BZD use and QoL?

To answer these questions, we combined and analysed data from two previously published studies on the same patient sample at the University Psychiatric Clinics of Basel, Switzerland [7, 25]. The data were collected in an outpatient centre providing traditional OAT [oral methadone, buprenorphine, and slow-release oral morphine (SROM)] and a centre specialised in heroin-assisted treatment [injectable diacetylmorphine (DAM)]. At the time of data collection, 360 patients received either traditional OAT or heroin-assisted treatment in these centres and were therefore potential participants of the initial studies. In total, 315 patients were approached and asked whether they were interested in participation. This was done separately for both studies and 39.2% of the overall population agreed to participate in both studies. In these centres, the dose of prescribed opioids is typically stable and co-use of illicit substances does not lead to treatment exclusion. Inclusion criteria were the same for both previous studies and comprised presence of OUD and having the ability to give informed consent. Convenience sampling was used, and participation was completely voluntary. Only patients who participated in both studies (i.e., data on BZD use and QoL was available) were included in the current study (n = 141).

### Participant-rated measurements

The Symptom Checklist-27 (SCL-27) is a validated modification of the widely used Symptom Checklist-90-R and screens for psychiatric symptoms and psychological distress. Each of the six subscales (depressive, dysthymic, vegetative, agoraphobic, sociophobic symptoms and symptoms of mistrust) consists of 4–6 items that are rated on a 5-point Likert scale. It allows the calculation of the Global Severity Index (GSI), which is a global composite score. The instrument has been validated and shows good internal consistency [26]. It is commonly utilised in addiction research and has been validated and recommended for the use in psychiatric populations [27, 28].

The Allgemeine Depressionsskala (ADS-L) is the German version of the Center for Epidemiologic Studies Depressions Scale [29]. The ADS-L is a 20-item self-report instrument of depressive state. Each item is rated on a 4-point Likert scale and the instrument has been validated for use in general and clinical populations [30] and has previously been employed in research on opioid dependent populations [31, 32].

The German version of the Lancashire Quality of Life Profile (LQOLP) was used to assess QoL [33]. The instrument shows satisfactory reliability and validity [34]. It consists of 10 domains for work and education, leisure, religion, finances, living situation, safety, family relations, social relations, health (including mental health), and overall life satisfaction. A modification by Giacomuzzi et al. (2001) includes an additional domain regarding satisfaction with treatment for substance use disorder [35]. Items are rated on 7-point Likert scales (1 = completely dissatisfied; 7 = completely satisfied). A comprehensive measure of total QoL was obtained by summing up all 11 LQOLP domains—a procedure which has shown acceptable reliability in previous studies [36, 37].

BZD use was assessed through a self-report questionnaire, which was designed by a group of clinically experienced psychiatrists and psychologists. Lifetime BZD use was defined as self-report of ever having used BZD or a positive urine toxicology test. Prolonged BZD use was defined as self-report of having used BZD several times a week for a period of at least two months in the past 5 years. Current BZD use was defined as testing positive for BZD in the urine toxicology test or self-reporting use in the past 30 days. Former users were defined as those that reported lifetime use but no current use as defined by urine toxicology results or self-report. More details about the BZD questionnaire can be found in a previously published study [15].

### Other measures

Immunoassay urine toxicology testing was performed on the day of study participation to confirm self-reported data. The analysis included BZD, amphetamines, barbiturates, tetrahydrocannabinol, cocaine, tricyclic antidepressants (TCA), methadone, morphine, and buprenorphine. When analysing additional substances found in urine toxicology testing, all except opioid agonist medication, BZD, and TCA were included. Alcohol use was assessed through self-reports. The electronic patient file provided data on age, gender, current prescribed medication, treatment duration and psychiatric diagnoses (ICD-10).

### Medication conversion

Opioid agonist doses were converted into methadone equivalent doses through the following scheme: injectable diacetylmorphine:methadone 4:1, oral diacetylmorphine:methadone 8:1, slow-release oral morphine:methadone 8:1, buprenorphine:methadone 1:6, and codeine:methadone 12:1 [38].

BZD doses were converted into diazepam equivalent doses through the following scheme: alprazolam:diazepam 1:10, bromazepam:diazepam 1:1.6, clonazepam:diazepam 1:5, flunitrazepam:diazepam 1:20, flurazepam:diazepam 3:1, lorazepam:diazepam 1:5, midazolam:diazepam 1:1.3, and oxazapam:diazepam 3:1 [39, 40].

### Statistical analysis

Statistical analysis was performed with SPSS version 28 (IBM). Missing data was substituted by the median of the respective variable in cases where less than 10% of answers were missing. Level of significance was set at p < 0.05 for all calculations. Kolmogorov–Smirnov tests were used to assess data distribution. Mann–Whitney-U tests and effect sizes r were calculated to test for group differences between continuous variables. Pearson’s correlation coefficient was calculated to test the linear relationship between continuous variables. Multivariable linear regression models were calculated to identify variables predicting total QoL scores. Chi-squared tests were performed to test for frequency differences of BZD use patterns between oral and injectable OAT patients.

## Results

### Sample description

The mean age in the sample (n = 141) was 42 years (SD = 7.2) and the majority were male (66.0%; n = 93). Lifetime BZD use was found in 88.7% of participants (n = 125). A total of 82 were currently using BZD (58.2%) and 61 reported prolonged BZD use (43.6%). Lifetime users who had negative urine toxicology test results and reported no past 30-day BZD use (n = 43; 30.5%) were considered former users. There were no differences in BZD use pattern frequency between patients injecting DAM and patients receiving oral medication. Sample characteristics are provided in Table 1.

**Table 1:** Sample characteristics (n = 141)

	n (%)	M (SD)	MD (min–max)
Sex			
Female	48 (34.0)		
Male	93 (66.0)		
Age		42.0 (7.2)	
Age of first opioid use		18.9 (3.4)	
Opioid dose (methadone equivalents)		108.9 (53.5)	100.0 (15–300)
Duration of current OAT			
< 1 year	17 (12.1)		
1–4 years	36 (25.5)		
5–9 years	34 (24.1)		
> 10 years	54 (38.3)		
Opioid agonists prescribed^a^			
Methadone	76 (53.9)		
SROM	22 (15.6)		
DAM	79 (56.0)		
Buprenorphine	7 (5.0)		
Lifetime BZD use	125 (88.7)		
Prolonged BZD use^b^	61 (43.6)		
Current BZD use	82 (58.2)		
Former BZD use	43 (30.5)		
Age at first BZD use		22.1 (7.2)	
BZD dose (daily diazepam dose equivalents)		24.0 (29.1)	20.0 (0–210)
Current use of other substances^c^ (excluding prescription opioids, BZD, and TCA)			
None	56 (39.7)		
1	63 (44.7)		
2	18 (12.8)		
3	2 (1.4)		
Missing	2 (1.4)		
HIV seropositive	10 (7.1)		
HCV seropositive	97 (68.8)		
Non-substance related mental disorders			
None	47 (33.3)		
1	58 (41.1)		
2	34 (24.1)		
3	2 (1.4)		

^a^Combinations possible; ^b^missing data in one participant (n = 140); ^c^urine toxicology testing; OAT: opioid agonist treatment; SROM: slow-release oral morphine; DAM: diacetylmorphine; BZD: benzodiazepine; TCA: tricyclic antidepressants; HIV: human immunodeficiency virus; HCV: hepatitis c virus

### BZD use and QoL

An overview of the bivariate analysis results as well as LQOLP domain scores grouped by BZD use patterns is presented in Table 2.

**Table 2:** Domain scores and group differences and in LQOLP domains as determined by the Mann–Whitney-U test

Domain (scale range)	Total sample (n = 141)			Lifetime BZD use (n = 125)^a^			Prolonged BZD use (n = 61)^a^			Current BZD use (n = 82)^a^			Former BZD use (n = 43)^b^		
	p	r	M (SD)	p	r	M (SD)	p	r	M (SD)	p	r	M (SD)	p	r	M (SD)
Life overall (1–7)	–	–	4.29 (1.30)	0.019*	− 0.20	4.20 (1.27)	0.008**	− 0.22	3.98 (1.28)	0.004**	− 0.24	4.04 (1.28)	0.033*	0.19	4.51 (1.20)
Work and education (1–7)	–	–	4.12 (1.68)	0.015*	− 0.21	3.98 (1.64)	0.131	–	3.82 (1.59)	0.061	–	3.86 (1.64)	0.355	–	4.23 (1.64)
Leisure (1–7)	–	–	4.05 (1.44)	0.083	–	3.96 (1.42)	0.127	–	3.83 (1.28)	0.020*	− 0.20	3.81 (1.40)	0.076	–	4.27 (1.44)
Religion (1–7)	–	–	5.02 (1.56)	< 0.001***	− 0.33	4.85 (1.57)	< 0.001***	− 0.40	4.32 (1.71)	0.011*	− 0.26	4.67 (1.70)	0.186	–	5.19 (1.25)
Finances (1–7)	–	–	3.29 (1.60)	0.237	–	3.22 (1.53)	0.111	–	3.04 (1.43)	0.010*	− 0.22	2.98 (1.40)	0.019*	0.21	3.68 (1.67)
Living situation (1–7)	–	–	4.87 (1.33)	0.314	–	4.82 (1.34)	0.263	–	4.74 (1.29)	0.035*	− 0.18	4.68 (1.33)	0.062	–	5.10 (1.33)
Safety (1–7)	–	–	5.33 (1.26)	0.077	–	5.26 (1.29)	0.219	–	5.22 (1.23)	0.072	–	5.16 (1.34)	0.249	–	5.44 (1.17)
Family relations (1–7)	–	–	4.47 (1.44)	0.162	–	4.42 (1.41)	0.430	–	4.40 (1.46)	0.108	–	4.33 (1.50)	0.234	–	4.59 (1.22)
Social relations (1–7)	–	–	4.90 (1.22)	0.004**	− 0.24	4.79 (1.21)	0.002**	− 0.26	4.54 (1.21)	0.053	–	4.71 (1.26)	0.407	–	4.95 (1.11)
Health (1–7)	–	–	4.58 (1.20)	0.014*	− 0.21	4.49 (1.18)	< 0.001***	− 0.34	4.12 (1.10)	0.028*	− 0.18	4.41 (1.15)	0.209	–	4.64 (1.24)
Route of administration (1–7)	–	–	5.57 (1.29)	0.508	–	5.54 (1.32)	0.106	–	5.36 (1.40)	0.423	–	5.46 (1.42)	0.561	–	5.70 (1.10)
Total QoL (7–77)	–	–	49.03 (9.29)	0.004**	− 0.25	48.11 (8.85)	< 0.001***	− 0.28	46.17 (7.72)	< 0.001***	− 0.29	46.67 (8.79)	0.017*	0.21	50.86 (8.41)

*Indicates p < 0.05; **indicates p < 0.01; ***indicates p < 0.001; ^a^compared to the rest of the sample; ^b^compared to lifetime users with current use

Patients with lifetime BZD use (n = 125) were significantly less satisfied with their life overall (U = 648.0, p = 0.019, r = − 0.20), their work and education (U = 627.5, p = 0.015, r = − 0.21), their religious life (U = 222.5, p < 0.001, r = − 0.33), their social relations (U = 566.5, p = 0.004, r = − 0.24), and their health (U = 623.0, p = 0.014, r = − 0.21) when compared to patients without lifetime BZD use.

Patients with prolonged BZD use (n = 61) were significantly less satisfied with their life overall (U = 1795.0, p = 0.008, r = − 0.22), their religious life (U = 675.5, p < 0.001, r = − 0.40), their social relations (U = 1689.0, p = 0.002, r = − 0.26), and their health (U = 1451.0, p < 0.001, r = − 0.34) when compared to patients without prolonged BZD use.

Patients with current BZD use (n = 82) as determined by urine toxicology testing were significantly less satisfied with their life overall (U = 1748.0, p = 0.004, r = − 0.24), their leisure (U = 1858.5, p = 0.020, r = − 0.20), their religious life (U = 870.0, p = 0.011, r = − 0.26), their financial situation (U = 1808.5, p = 0.010, r = − 0.22), their living situation (U = 1915.0, p = 0.035, r = − 0.18) and their health (U = 1896.0, p = 0.028, r = -0.18) when compared to patients without current BZD use.

Former BZD users were significantly more satisfied with their life overall (U = 1363.0, p = 0.033, r = 0.19) and their finances (U = 1313.0, p = 0.019, r = 0.21) when compared to current users. Compared to patients who had never used BZD in their lifetime, former users were significantly less satisfied with their religious life (U = 88.5, p = 0.005, r = − 0.43) and their social relations (U = 209.0, p = 0.019, r = − 0.30).

Total QoL scores were significantly lower in lifetime (U = 648.0, p = 0.019, r = − 0.20), prolonged (U = 1620.0, p < 0.001, r = − 0.28), and current users (U = 1608.5, p < 0.001, r = − 0.29) when compared to patients without the respective use pattern. Former BZD users had significantly higher total QoL scores when compared to current users (U = 1304.5, p = 0.017, r = 0.21), but no significant difference was found compared to patients without lifetime use (U = 247.5, p = 0.100). Effect sizes were weak to moderate for all group differences.

### Relationship between BZD dose and QoL

Out of 82 participants with current BZD use, 61 patients responded on BZD dose (74.4%). Pearson’s r was calculated to assess the linear relationship between total QoL score and BZD dose. No significant correlation was found between the two variables, r(59) = 0.01, p = 0.923.

### Linear regression

Multivariable linear regression models were calculated to analyse the association of BZD with total QoL corrected for possible confounders. Due to the intercorrelation between BZD use patterns, four separate models were calculated, and each included a different use pattern as independent variable. Furthermore, all models included additional variables possibly influencing QoL (age, sex, number of additional substances found in urine toxicology testing, depressive state as determined by the ADS-L, and psychiatric symptom load as determined by the GSI). Variables were controlled for multicollinearity by calculating variance inflation factors.

Lifetime BZD predicted a lower total QoL score and the model explained 53% of total QoL variance respectively (Table 3). In all models, female sex significantly predicted higher total QoL scores, and higher ADS-L scores significantly predicted lower QoL scores.

**Table 3:** Multivariable linear regression models

Variable	B	SE	t	p	95% CI
(Constant)	64.586	3.649	17.698	< 0.001	57.368 to 71.805
Age	− 0.051	0.076	− 0.672	0.503	− 0.201 to 0.099
Sex^a^	4.362	1.145	3.810	< 0.001***	2.097 to 6.626
No. of additional substances in urine	− .797	0.760	− 1.048	0.296	− 2.301 to 0.707
Psychiatric symptom load	− 1.281	1.450	− 0.883	0.379	− 4.150 to 1.588
Depressive state^b^	− 0.500	0.078	− 6.390	< 0.001***	− 0.655 to − 0.345
Lifetime BZD use	− 4.544	1.720	− 2.641	0.009**	− 7.946 to − 1.141
R^2^ = 0.548; adjusted R^2^ = 0.528; n = 139;					
(Constant)	61.449	3.429	17.919	< 0.001	54.665 to 68.232
Age	− 0.045	0.077	− 0.590	0.556	− 0.197 to 0.107
Sex^a^	4.354	1.159	3.756	< 0.001***	2.601 to 6.648
No. of additional substances in urine	− 0.444	0.778	− 0.570	0.570	− 1.982 to 1.095
Psychiatric symptom load	− 1.483	1.465	− 1.013	0.313	− 4.381 to 1.414
Depressive state^b^	− 0.497	0.080	− 6.239	< 0.001***	− 0.655 to − 0.340
Current BZD use	− 2.220	1.154	− 1.924	0.057	− 4.503 to 0.063
R^2^ = 0.537; adjusted R^2^ = 0.516; n = 139;					
(Constant)	61.161	3.460	17.677	< 0.001	54.317 to 68.006
Age	− 0.050	0.078	− 0.648	0.518	− 0.204 to 0.103
Sex^a^	4.487	1.175	3.819	< 0.001***	2.163 to 6.812
No. of additional substances in urine	− 0.570	0.783	− 0.728	0.468	− 2.118 to 0.978
Psychiatric symptom load	− 1.622	1.488	− 1.089	0.278	− 4.566 to 1.323
Depressive state^b^	− 0.503	0.081	− 6.243	< 0.001***	− 0.663 to − 0.344
Prolonged BZD use	− 1.058	1.185	− 0.893	0.373	− 3.401 to 1.285
R^2^ = 0.532; adjusted R^2^ = 0.510; n = 138;					
(Constant)	55.981	3.510	15.951	0.001	49.030 to 62.932
Age	0.044	0.077	0.563	0.574	− 0.110 to 0.197
Sex^a^	4.672	1.143	4.088	0.001***	2.408 to 6.935
No. of additional substances in urine	− 0.513	0.746	− 0.688	0.493	− 1.992 to 0.965
Psychiatric symptom load	0.824	1.489	.553	0.581	− 2.125 to 3.773
Depressive state^b^	− 0.612	0.084	− 7.293	0.001***	− 0.778 to − .446
Former BZD use	1.031	1.177	0.876	0.383	− 1.301 to 3.363
R^2^ = 0.563; adjusted R^2^ = 0.540; n = 123					

Dependent variable: total QoL score; ^a^male sex as reference; **indicates p < 0.01; ***indicates p < 0.001; ^b^as measured by the ADS-L

## Discussion

The present study adds to the body of literature on the specific association between BZD use patterns and QoL in patients receiving OAT. Bivariate analysis showed significantly lower total QoL scores for lifetime, current, and prolonged BZD users. However, when controlling for psychiatric symptom load and depressive state in linear regression, lifetime BZD use remained the only pattern significantly predicting lower QoL. This finding suggests that psychiatric comorbidity and the resulting symptom load play a larger role in diminishing the QoL in OAT patients than BZD use itself. Our results support the findings of Carpentier et al., (2009), who reported poorer QoL in OAT patients with dual diagnoses, without BZD/sedative use significantly influencing QoL [41, 42]. In contrast, a recent study on high-dose BZD users with or without OUD found no influence of psychiatric disorders and the authors concluded that BZD use per se exerts a negative influence on QoL [43]. Several explanations for the discrepancies are possible. First, our sample was in OAT, which has been shown to improve QoL [14]. Second, compared to Tamburin et al., our sample used lower doses overall. Third, we controlled for psychiatric symptom load, which may be more accurate than controlling for a psychiatric diagnosis, which may or may not be remitted at the time of study.

BZD-using individuals may suffer from an increased burden of psychiatric symptoms, in particular depression and anxiety. Illicit BZD use may then occur as self-medication rather than recreational use in search of a “high”. Likewise, BZD prescription may constitute a treatment attempt for comorbid psychiatric conditions, although not in line with current treatment guidelines for mental disorders that usually discourage long-term BZD prescription [44]. Use of illicit BZD may also occur in the context of an untreated and unstable BZD use disorder, equally having a negative impact on QoL that is less confounded by psychiatric symptoms. In our sample, most BZD-using patients had a long-term BZD prescription. Although we did not specifically assess this, these prescriptions are often within the context of an off-label maintenance approach and are common in Switzerland [20]. Clinical experience and literature, albeit scarce, shows that this approach is effective at stabilising BZD dependence [45]. Successful maintenance treatment of BZD dependence may then result in a reduction of the negative effects of BZD on QoL. Despite the relevance of BZD use in OAT, and relatively common long-term treatments with BZD, no double-blind placebo-controlled studies have addressed BZD maintenance treatment in OAT so far. Future studies should examine this issue.

These two possible mechanisms provide an explanation of both the high prevalence of BZD use in our sample as well as the link between BZD use and QoL. However, state of the art treatment of co-occurring mental disorders or BZD dependence does not necessarily require BZD prescriptions but rather targeted psycho- and pharmacotherapeutic approaches. Such an appropriate treatment of comorbid mental or psychiatric disorders would allow reducing BZD use and psychiatric symptom load alike, improving the QoL of opioid-dependent patients [46]. It may also allow a reduction of prescription as well as illicit BZD use, which has been linked to negative health outcomes and increased mortality [21]. Our results may be confounded by different indications of BZD prescriptions or motives for use, as they were not controlled for. Future studies should employ a prospective, longitudinal design to test the impact of targeted treatment on QoL and BZD use while also controlling for the underlying indication or intake motive.

The clinical importance of adequately treating psychiatric comorbidity in order to improve patient well-being is also illustrated by the association between depressive state and lower total QoL in all linear regression models from our study. Individuals with OUD are at high risk of developing depressive disorders, with reported lifetime prevalence rates of up to 75% [47, 48], adding even more weight to the effective treatment of comorbid disorders. While surely better than no treatment, the mere dispensing of medication is insufficient and integrated treatment should ideally be offered with OAT provision [49].

In summary, although psychiatric comorbidity is a well-known very common phenomenon in this patient population [50], real-world clinical practice still does not consistently apply appropriate treatment models which address this issue [51]. Integrated treatment models including psychosocial and psychiatric interventions are recommended by many guidelines but are still the exception, not the rule [52]. Our study underlines that meticulous diagnosis and treatment of co-occurring mental disorders in OAT is paramount to improve QoL in this patient population and may also help reduce BZD use.

### Strengths and limitations

Our study has some limitations. Firstly, our sample consisted of patients in OAT and our results may therefore not be representative for patients with OUD outside of treatment. Secondly, due to the method of recruitment (convenience sampling), we cannot rule out that participants systematically differ from non-participating patients. However, sex and age distribution were similar to the overall distribution in Swiss OAT. Thirdly, the cross-sectional nature of the study does not allow causal inference. Longitudinal studies investigating BZD use, psychopathology and QoL are necessary to further illuminate the complex relationship between these factors. Although well established in research, the ADS-L cannot discriminate whether measured symptoms are attributable to opioid use or depression, therefore decreasing its reliability in the studied population. One of the strengths of this study is the assessment and consideration of current psychological symptom distress. Furthermore, we provide data for patients in oral as well as injectable OAT, a group with particular vulnerabilities.

Notably, our study relied on the self-reporting of BZD use patterns in addition to urine toxicology testing. Concurrent use of non-prescribed substances is commonly sanctioned in OAT settings and can lead to treatment exclusion. Therefore, underreporting of substance use has been observed in this context [53]. However, it has also been demonstrated that self-reporting is reliable when their outcome is not coupled to negative consequences [54]. This study was conducted in treatment settings in which concurrent substance use does not lead to negative consequences or puts patients at risk of treatment exclusion.

## Conclusions

Lifetime, current, and prolonged use of BZD in OAT is associated with reduced QoL. Because the significant association only persisted for lifetime use when controlling for psychiatric symptoms in the multivariable linear regression models, our results suggest that this finding is an effect of psychiatric symptoms commonly occurring in OAT patients rather than of BZD use per se. Treatment of co-occurring mental disorders, particularly depression, is paramount to reduce psychiatric symptom load and improve QoL in this patient population.

## Data Availability

The datasets used and/or analysed during the current study are available from the corresponding author on reasonable request.

## Abbreviations

BZD: Benzodiazepine
OUD: Opioid use disorder
OAT: Opioid agonist treatment
QoL: Quality of life
SROM: Slow-release oral morphine
DAM: Diacetylmorphine
SCL-27: Symptom Checklist 27
GSI: Global Severity Index
ADS-L: Allgemeine Depressionsskala-Lang
LQOLP: Lancashire Quality of Life Profile
TCA: Tricyclic antidepressant
